# The Impact of COVID-19 on Medical Education

**DOI:** 10.7759/cureus.7492

**Published:** 2020-03-31

**Authors:** Meganne N Ferrel, John J Ryan

**Affiliations:** 1 Medicine, University of Utah School of Medicine, Salt Lake City, USA; 2 Cardiology, University of Utah, Salt Lake City, USA

**Keywords:** pandemic, usmle, graduation, coronavirus, graduate medical education, acgme

## Abstract

In the wake of the novel coronavirus (COVID-19) pandemic, it is abundantly clear to all the necessity of studying the pathology and widespread health consequences associated with the virus. However, what is much less clear is the impact of COVID-19 on medical education. Already, faculty and medical students are grappling with the changes that have been made and attempting to consolidate these with their plan of career development. Changes that may seem relatively minor in comparison to the global pandemic have the potential to be drastic turning points in the career progression of many. As not much is known regarding the long-lasting impact of COVID-19 on medical education, it is therefore also necessary to record and study the full impact of the changes being made.

The path to entering a successful residency has been predictable for the last few years - do well on Step 1, give conference presentations, go the extra mile in clerkships and shadowing opportunities, and have meaningful non-academic extracurricular activities - all of which designed to best demonstrate a student's knowledge, persistence, collaborative spirit, and dedication to medicine. This trajectory has been changed with COVID-19 disrupting routines in hospitals, medical schools and beyond. The replacement of in-person classes with online equivalents is an obvious necessity at this time but creates a loss of collaborative experiences that has the potential to be a significant detriment to education. Likewise, the cancellation of clerkships, which are necessary for both skill acquisition as well as for relationship building, is a serious issue which students and medical schools must now resolve. Many medical students have also lost the opportunity for personal development through conference presentations. These presentations play a large role in distinguishing applicants during the residency application process, and therefore these lost opportunities have the potential to be a serious detriment to medical students’ career trajectory. While implementing technology to help resolve these issues is a unique way to help students to develop these skills, it is now necessary for medical students to demonstrate the same set of skills which they would have previously in a completely new and innovative manner.

Persistence and adaptability during this time of challenge are attributes that medical students can demonstrate more readily. While every student has a personal story of how COVID-19 has impacted their education, there is no question that the impacts of COVID-19 will be felt on an extensive level. The panic in the community is palpable, and many are confused by how to proceed in the wake of COVID-19. This is no different for medical students and faculty and the questions that arise regarding medical education and their future careers.

## Editorial

Studying world questions and preparing classes for Unites States Medical Licensing Examination (USMLE) Step 1 does not adequately equip a person for when family and friends ask, “What do you know about coronavirus?”. Having a mental picture of a Sketchy Medical image or being able to teach that, “It causes a respiratory illness”, while having in the back of one’s head that it is a positive sense RNA enveloped virus with a helical capsid is not particularly useful. However, over the course of the past few weeks, for medical school faculty and medical students, the novel coronavirus (COVID-19) has undergone a transformation from an obscure study tool picture to something that is a turning point in many careers.

Initially, before the COVID-19 had become widespread in the United States, many students had not thought much about how it was going to affect them. Although students care about the wellbeing of others around them, and following events in China from afar, while studying for Steps or boards, medical students have comparatively little human interaction and therefore little risk. Additionally, it was not particularly clear at first to what degree US medical students should be concerned about COVID-19. While some sources were claiming that it is was being blown out of proportion, others were raising the alarm about the serious nature of this illness. Now, COVID-19 is disrupting routines in hospitals, medical schools, and beyond. The health consequences and mortality are already catastrophic and medical education is being adapted due to this worldwide threat. We will not know the full impact of COVID-19 on medical education for quite some time. As such it will be important to record and study the full extent of the changes in medical education being made in response to this national emergency so as to clarify how we recover from this pandemic.

One of the most immediate changes introduced has been the broad cancelling of in-person medical classes, with most being replaced by recorded lectures or live-streams. Admittedly, a common trend of medical students across the country during didactic years is to primarily use outside resources for Step preparations and to watch school lectures after it has already been presented on 2x speed. Therefore, by cancelling classes, many medical students in their didactic years may perceive little change in their study schedule, but the loss of collaborative experiences has the potential to become a detriment to education and is worth studying. In addition, many faculty have been emphasizing the irreplaceable value of attending class in-person, lauding the real-time feedback and back-and-forth that develop in class that are hard to replicate in online forums. Now with classes being moved online, faculty are caught speaking out of both sides of their mouth. Standards to mimic these interactive discussions online will need to be studied in order to ensure that the online experience is adequate in preparing our students for clinical clerkships and beyond. One technique that demonstrates this collaborative environment currently being implemented include small group meetings in an online forum. The cancellation of classes will present a challenge to re-engage students within the community spirit of medical school once restrictions are lifted. Interactive learning groups, such as small group case-based learning and team-based learning can continue during the COVID-19 pandemic through webinars and teleconferences and perhaps provides an insight into how medical school will look going forward.

For a variety of reasons, many schools have also cancelled clinical clerkships. One reason is to flatten the curve, with the goal of minimizing personal interactions to mitigate and contain the spread of COVID-19. Another reason is to decrease the risk of exposure for medical students, which is an understandable concern, although many students are willing to put themselves at risk and as such can be frustrated by these decisions. Finally, with the current lack of personal protective equipment, cancelling clerkships is a necessity in order to ensure that healthcare workers are adequately able to protect themselves during this pandemic. In order to learn from this pandemic, it will be important for medical schools to share best practices as to how they are addressing the cancellation of clerkships in order to ensure that students progress forward within medical school and ultimately satisfy graduation requirements on-time.

Another way in which COVID-19 has affected medical education is the cancelling of medical conferences. These conferences, and the associated presentations that medical students give, are essential to building up medical students resumes and applications for residency. Although minor in the context of the global pandemic, it raises the question on how to account for these changes and adapt as a trainee. As a student, one has to learn how to balance the status of being a learner planning out one’s career, with the privilege of being a future health care provider who can valuably contribute to the health crisis at this time. As many medical students now will miss out on the valuable experiences of presentations, clinical rotations, and collaborative experiences - standards which helped previous generations become future doctors - the question arises of how students will evolve and integrate themselves into the medical community. When considering the reasons why conference presentations and extracurricular activities become so central to residency applications, it is important to recall what they actually represent for each student [1]. Therefore, we must consider how medical students can develop and demonstrate skills such as knowledge, determination, and collaboration and best prepare for the careers ahead of them in the face of these recent changes. Since students feel that they are missing out on the opportunities that these in-person conferences and presentations provide, there is a growing demand for organizing online conferences. This has been met with recommendations for the designing and organizing such events [2]. The younger generations in medical school are perhaps best equipped to integrate technology and webinars into health care delivery and sharing medical knowledge in innovative, online settings.

Implementing technology into medical education in a unique way will allow students to develop collaborative skills and improve adaptability. Navigating the challenges associated with remote collaboration with their peers sets up a unique parallel and practice to what interprofessional cooperation and telemedicine could look like in our future careers. Students who are better able to adapt to this unique situation of COVID-19 will show their ability to think outside of the box and alter pre-conceived notions of how medicine should be practiced. Therefore, this demonstrates a student’s adaptability and innovation. Students must be innovative in devising ways to exhibit their skills, work ethic, teamwork, and dedication to research. Persistence and adaptability during this time will be an attribute more readily viewed in the face of these new challenges and the innovative approaches to addressing these difficulties.

The effect of COVID-19 has begun to have an impact in other aspects of students’ career progression and lives. The panic in the community is palpable, and many are confused by how to proceed in the wake of COVID-19. Medical students are asking what actions they can take to aid their communities and to decrease the total potential for damage [3]. Medical students have the capacity to reinforce the recommendations as outlined by the Centers for Disease Control and Prevention (CDC) and our legislative bodies. Despite being instructed to stay at home for now, they can also contribute to the effort. Many faculty are being called in to work and with schools and daycares being closed, this presents unique challenges of covering children and work. Within some medical schools, students have coalesced to provide childcare and home care services for faculty [4]. Additionally, these times away from clinical rotations do present opportunities for students to participate in academic writing, although these can be difficult to find with faculty being drawn to significant amount of clinical work. Most students would rather remain at the frontlines, and at least in some medical schools, there is consideration to graduate medical students early to help provide additional staffing in general practitioners’ clinics and in the homes of elderly people [5].

Regardless of the unique nuances of each medical student’s situation, every student will face difficulties that have arisen due to the widespread effects of the COVID-19 pandemic. COVID-19 now has a face for every student - in more than just a Sketchy image - that will be carried throughout their careers and for the rest of their lives. As students and faculty adapt during this pandemic, it will be important to study the extent to which the changes currently being introduced in response to COVID-19 impact medical education overall, as well as medical student career progression, personal health and safety.
